# Latent microbial reactivation and immune dysregulation in sarcoidosis: bridging pathogenesis and precision therapeutics

**DOI:** 10.3389/fmed.2025.1625915

**Published:** 2025-08-15

**Authors:** Michiru Sawahata, Keisuke Uchida, Asuka Furukawa, Yoshinobu Eishi, Makoto Maemondo

**Affiliations:** ^1^Department of Respiratory Medicine, Jichi Medical University, Tochigi, Japan; ^2^Division of Surgical Pathology, Institute of Science Tokyo Hospital, Tokyo, Japan; ^3^Department of Human Pathology, Graduate School of Medical and Dental Sciences, Institute of Science Tokyo, Tokyo, Japan

**Keywords:** sarcoidosis, cutibacterium acnes, latent intracellular infection, reactivation, regulatory T-lymphocytes, immune tolerance, granulomatous inflammation, precision medicine

## Abstract

Sarcoidosis, a systemic granulomatous disease of unknown etiology, is characterized by the formation of non-caseating granulomas affecting multiple organs. Accumulating evidence implicates *Cutibacterium acnes* (*C. acnes*; formerly *Propionibacterium acnes*) as a potential microbial trigger. The consistent detection of *C. acnes* within sarcoid granulomas, along with associated Th1-polarized immune responses, indicates that latent intracellular persistence and reactivation of this commensal bacterium may drive granulomatous inflammation. This bacterium can persist intracellularly within macrophages and dendritic cells and, upon reactivation, may induce Th1/Th17-dominant immune responses in genetically and immunologically susceptible individuals. Immune dysregulation, including deficient *C. acnes*-specific regulatory T cell (Treg) responses, may underlie the unchecked effector activity that sustains inflammation. Enhanced *C. acnes*-specific T-cell reactivity, including elevated interferon-γ and interleukin-2 production, is observed in some patients, supporting this hypothesis. Although direct evidence for *C. acnes*-specific Tregs and antigen-specific T-cell responses is limited, immune dysregulation involving impaired tolerance is thought to contribute to the heterogeneity of sarcoidosis, which ranges from spontaneous remission to chronic fibrotic progression. Recent advances in diagnostic tools, including *P. acnes*-specific monoclonal antibody immunostaining and T-cell assays specific to *C. acnes*, offer promising approaches for detecting microbial involvement. These developments highlight the importance of etiology-driven treatment strategies. As sarcoidosis likely comprises a spectrum of underlying causes, etiology-specific interventions are particularly warranted upon the identification of a defined trigger, such as *C. acnes*. This review explores the potential pathogenesis of sarcoidosis, focusing on latent microbial reactivation, immune dysregulation, and their diagnostic and therapeutic implications, and highlights opportunities for precision medicine.

## 1 Introduction

Sarcoidosis is a systemic granulomatous disorder of unknown etiology, characterized by the development of non-caseating epithelioid cell granulomas affecting multiple organs, most commonly the lungs, lymph nodes, skin, and eyes (1). Despite extensive research, the precise etiology remains unclear. Two major hypotheses have been proposed: one involving autoimmunity, and the other implicating persistent microbial antigens as triggers (2, 3). The latter is supported by the frequent detection of microbial DNA in sarcoid lesions, including *Mycobacterium tuberculosis* (MTB) (4, 5) and *Cutibacterium acnes* (*C. acnes*; formerly *Propionibacterium acnes*) (6, 7).

Although sarcoidosis likely encompasses diverse etiologies, *C. acnes* has emerged as a leading candidate due to its consistent detection within sarcoid granulomas across pulmonary and extrapulmonary sites (8–12). In addition to its localization within sarcoid granulomas, heightened *C. acnes*-specific Th1 immune responses, characterized by increased interferon (IFN)-γ and interleukin (IL)-2 production, are observed in the peripheral blood and bronchoalveolar lavage (BAL) cells of patients with sarcoidosis, supporting its potential role as a causative antigen (13–18). *C. acnes* is a common skin and mucosal commensal bacteria that can persist intracellularly within macrophages and dendritic cells, thereby evading immune clearance and sustaining antigenic stimulation (19–21). Together, these findings support a pathogenic model wherein latent intracellular *C. acnes* undergoes reactivation in genetically and immunologically susceptible individuals, triggering a Th1-skewed immune response and contributing to granulomatous inflammation (22).

Sarcoidosis has long been regarded as an enigmatic disease, straddling the boundaries between autoimmunity, chronic infection, and environmental hypersensitivity (23–25). Although numerous infectious and environmental agents have been proposed as potential triggers—including MTB and various fungi among microbial candidates, and inorganic particulates among non-infectious candidates—*C. acnes* is the only microbial agent that has been successfully isolated from sarcoid lesions (26–28). This unique distinction, along with its consistent detection within sarcoid granulomas and reproducible evidence of heightened *C. acnes*-specific Th1 immune responses, has renewed interest in *C. acnes* as a credible etiologic agent (29).

Despite the unique evidence supporting *C. acnes* involvement in sarcoidosis, the clinical and pathologic variability of sarcoidosis suggests that multiple etiologic triggers are involved. Reflecting this heterogeneity, sarcoidosis is now widely considered a syndrome rather than a single disease entity, defined by a shared granulomatous pathology that may result from diverse underlying triggers (30). This etiologic complexity suggests that different persistent antigens activate a common immunopathologic pathway in genetically and immunologically susceptible individuals. While such heterogeneity may explain the clinical and pathologic variability observed in sarcoidosis, each proposed trigger requires rigorous evaluation. For each individual patient, identifying the causative factor is essential for accurate diagnosis and effective treatment.

Immune dysregulation is a hallmark of sarcoidosis and includes Th1/Th17 dominance and impaired immune regulatory mechanisms, such as deficient regulatory T cell (Treg) responses (31, 32). This immunologic imbalance may contribute to the development and persistence of granulomatous inflammation, potentially bridging the microbial and autoimmune hypotheses (33). Genetic susceptibility, particularly major histocompatibility complex (MHC) class II allele variants, has also been associated with both disease onset and progression, suggesting that immune regulation by the host, rather than microbial presence alone, critically influences disease outcomes (34, 35). Given the interplay between microbial persistence and host immune regulation, clarifying how latent *C. acnes* is reactivated and escapes immune control in genetically and immunologically susceptible individuals may illuminate key aspects of the etiopathogenesis of sarcoidosis.

In this review, we propose an integrated pathogenic model in which latent *C. acnes* infection undergoes intracellular reactivation in predisposed individuals, driving immune dysregulation and granulomatous inflammation. We synthesize emerging immunopathologic findings, highlight failures of immune tolerance toward this commensal bacterium, and discuss the diagnostic and therapeutic implications for advancing precision medicine in sarcoidosis.

## 2 *C. acnes* as a cause of sarcoidosis

### 2.1 Challenges in identifying causative agents of granulomatous diseases

Granulomatous diseases are defined by the presence of granulomas, which are structured aggregates of immune cells, primarily macrophages, that develop in response to persistent, poorly degradable substances. Granulomas serve as a host defense mechanism to locally sequester these indigestible materials, especially when they cannot be effectively eliminated. Granulomas, however, may also cause chronic inflammation and tissue damage (36, 37). A key principle in understanding the pathology of granulomas is that the causative agent must be detectable within the granuloma or must have been present during its formation, underscoring the role of sustained antigenic stimulation (38, 39).

Granulomas are generally categorized into two types. Foreign body granulomas develop in response to inert materials and are characterized by macrophage fusion into multinucleated foreign-body-type giant cells, typically without substantial activation of the adaptive immune system (40). In contrast, epithelioid cell granulomas are induced by highly antigenic stimuli, leading to macrophage differentiation into epithelioid cells and multinucleated Langhans-type giant cells. This process is accompanied by pronounced activation of the adaptive immune system, particularly the recruitment and activation of antigen-specific T cells that orchestrate granuloma formation and maintenance (41).

The formation of epithelioid cell granulomas typically proceeds through sequential stages: macrophage recruitment, differentiation into epithelioid and multinucleated giant cells, and cytokine-mediated structural organization. Over time, granulomas may evolve into fibrotic or necrotic lesions, with necrosis being particularly prominent in tuberculosis due to tumor necrosis factor alpha (TNF-α)–mediated apoptosis (42–44). From a diagnostic perspective, recognition of these histologic patterns, particularly the identification of the causative agent within granulomas, is essential for accurately diagnosing granulomatous diseases. This is especially relevant for disorders of unknown etiology, such as sarcoidosis, where antigen detection can guide differential diagnosis and inform therapeutic decisions (38).

According to established diagnostic criteria for sarcoidosis, a tissue biopsy must show non-necrotizing granulomas and exclude other potential causes of granulomatous inflammation, such as infectious or environmental agents (45). From a histopathological perspective in sarcoidosis, it remains important to consider alternative infectious agents—such as fungi (e.g., *Histoplasma* spp.), other bacterial species (e.g., *Nocardia* spp., *Actinomyces* spp.), and even viruses—as well as non-infectious triggers including inorganic dusts and environmental antigens. These factors can vary by geographic region, which may affect the likelihood of detecting these agents in clinical samples and contribute to uncertainty in diagnosing sarcoidosis.

Sarcoidosis patients often exhibit heightened immune responses to mycobacterial antigens such as early secreted antigenic target-6, with elevated IFN-γ and TNF-α levels indicating Th1 polarization (46–48). Several findings, however, challenge the role of MTB as the causative agent. QuantiFERON-TB Gold assays do not show increased positivity in sarcoidosis, and acid-fast staining, cultures, and immunohistochemistry have failed to detect MTB in sarcoid granulomas (48–51). Although detection of MTB DNA by polymerase chain reaction (PCR) has yielded inconsistent results—positive in some studies and negative in others—these findings may reflect latent infection, particularly in tuberculosis-endemic regions, rather than true etiologic involvement (4, 52). Furthermore, anti-mycobacterial therapy shows limited efficacy in sarcoidosis, whereas immunosuppressants such as corticosteroids are typically effective (53, 54). Although tuberculosis and sarcoidosis are globally recognized to share overlapping clinical and radiologic features, distinguishing between them is particularly challenging in tuberculosis-endemic regions (55). Nevertheless, the failure to reproducibly identify MTB in sarcoid granulomas, despite extensive investigation, supports the view that MTB is unlikely to be the primary cause.

### 2.2 Detection of *C. acnes* within sarcoid granulomas

The search for a causative agent in sarcoidosis has been guided by two key pathologic principles: the agent should be present in sarcoid lymph nodes, as demonstrated by the Kveim reaction (56), and it should also be localized within granulomas that develop in response to persistent antigens. Based on these principles, we employed an immunopathologic strategy to directly identify candidate microbes within granulomatous lesions.

**FIGURE 1:**
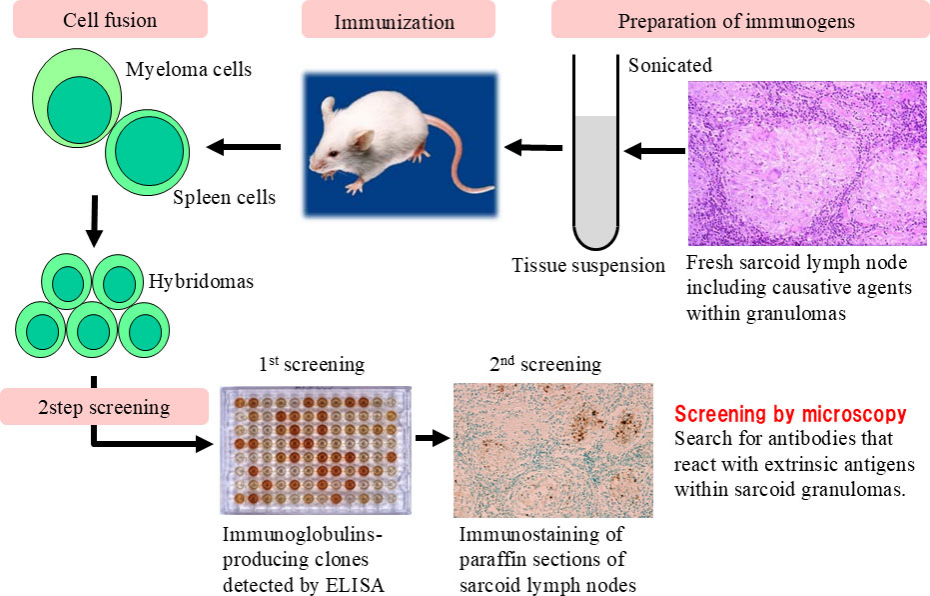
Generation of monoclonal antibodies against extrinsic antigens in sarcoid granulomas. This schematic outlines the production of monoclonal antibodies targeting microbial antigens in sarcoid granulomas. Fresh sarcoid lymph nodes were sonicated to prepare immunogenic tissue suspensions for mouse immunization. Spleen cells were fused with myeloma cells to generate hybridomas. Screening involved (1) enzyme-linked immunosorbent assay for antibody-producing clones and (2) immunostaining of paraffin-embedded sarcoid lymph node sections. Granuloma-specific antibody reactivity was confirmed microscopically. This strategy led to the identification of the SG5 antibody, which specifically bound to extrinsic antigens within sarcoid granulomas, providing a valuable tool for detecting microbial components potentially involved in granulomatous inflammation.

**FIGURE 2:**
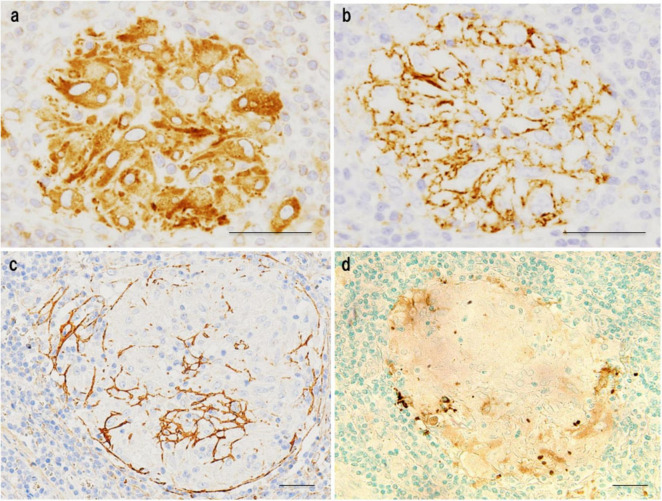
Immunohistochemical analysis of granuloma-reactive antibodies from mice immunized with fresh sarcoid lymph node tissue. Representative staining patterns of monoclonal antibodies showing distinct reactivity within sarcoid granulomas: **(a)** Cytoplasmic staining of epithelioid cells, suggesting recognition of intracellular components. **(b)** Membranous staining of epithelioid cells, indicating association with surface structures. **(c)** Staining of reticulin fibers, suggesting interaction with the extracellular matrix. **(d)** Staining of an extrinsic antigenic substance within the granuloma by the SG5 antibody, which specifically reacts with the culture supernatant of *C. acnes* but not with that of *M. tuberculosis*, indicating selective recognition of an organism-derived antigen. Scale bar: 50 μm. All images are original.

To explore potential antigenic targets within sarcoid granulomas, we first immunized mice with homogenized sarcoid lymph node tissue to generate monoclonal antibodies (Figure 1). Among these, one antibody (SG5) produced a characteristic dot-like staining pattern within granulomas (Figure 2) and reacted specifically with the culture supernatant of *C. acnes*, but not that of MTB, indicating *C. acnes* as a plausible antigenic candidate (57). To further validate this association, we immunized mice with *C. acnes* lysate, which led to development of the *P. acnes*-specific monoclonal antibody (PAB). This antibody selectively recognizes the cell membrane-bound lipoteichoic acid (LTA) of *C. acnes* and shows no cross-reactivity with other bacterial species, including MTB (8).

**FIGURE 3:**
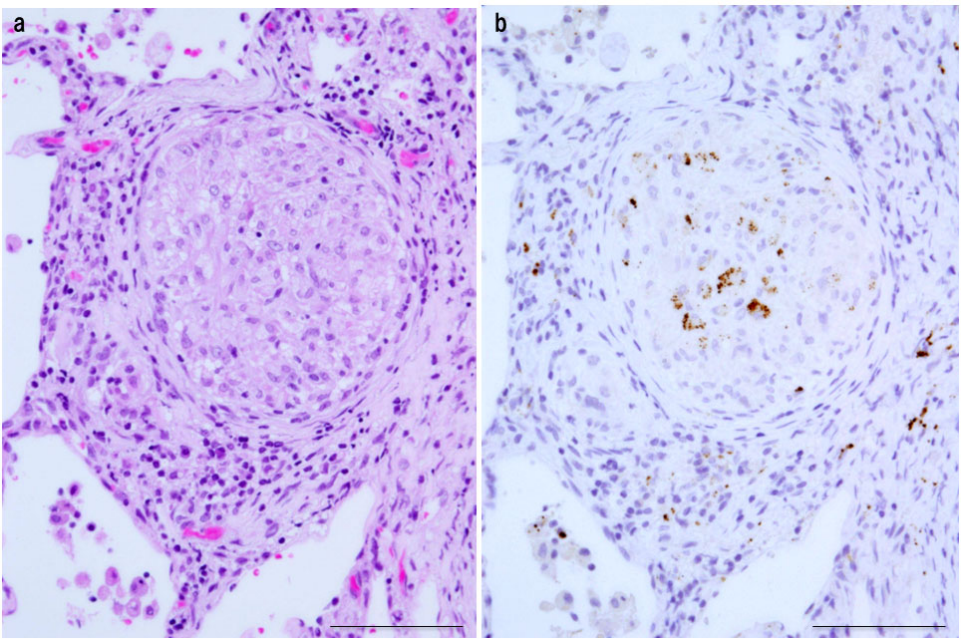
Detection of *C. acnes* in pulmonary sarcoid granulomas by *P. acnes* specific monoclonal antibody (PAB) antibody immunohistochemistry. **(a)** HE staining shows a well-formed non-caseating epithelioid granuloma in pulmonary tissue from a patient with sarcoidosis. **(b)** Immunostaining of a serial section with the *C. acnes*-specific PAB antibody, which targets membrane-bound lipoteichoic acid, reveals clustered brown signals within epithelioid cell cytoplasm, suggesting intracellular proliferation. Faint or fragmented signals indicate partial degradation during intracellular digestion. Scale bar: 100 μm. All images are original and previously published (8).

**FIGURE 4:**
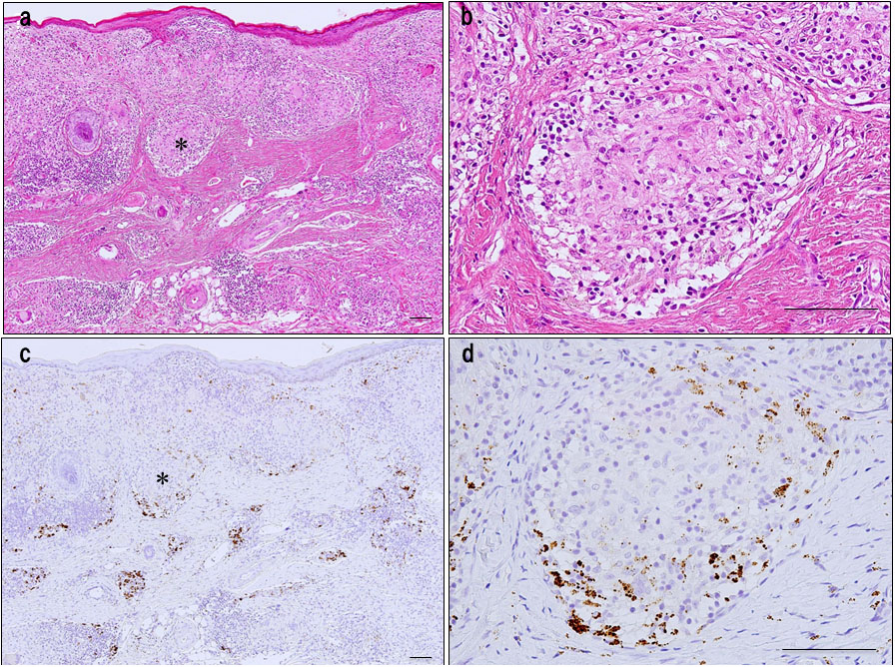
Detection of *C. acnes* in cutaneous sarcoidosis by *P. acnes* specific monoclonal antibody (PAB) antibody immunohistochemistry. **(a,b)** HE staining of a skin biopsy shows numerous non-caseating epithelioid granulomas with peripheral lymphocytic infiltration in the dermis [**(a)**, asterisk]. High-power view highlighting dense lymphocytic infiltration around the granuloma **(b)**. **(c,d)** Immunostaining of serial sections with *C. acnes*-specific PAB antibody shows strong signals at the granuloma periphery with lymphocytic infiltration [**(c)**, asterisk; **(d)**]. Central areas display faint, granular staining, suggesting partial degradation of bacterial components. Scale bar: 100 μm. All images are original and previously published (51).

*Propionibacterium acnes*-specific monoclonal antibody immunostaining demonstrated the presence of small, round *C. acnes*-positive bodies within epithelioid cells and multinucleated giant cells of sarcoid granulomas (Figure 3). These signals were most prominent at the granuloma periphery, where lymphocytic infiltration was dense (Figure 4), suggesting active immune recognition of bacterial antigens. In contrast, central regions exhibited only faint granular staining, a pattern consistent with antigen degradation in more mature granulomas. Occasionally, dense clusters of PAB-positive bodies were observed, implying possible intracellular proliferation of *C. acnes* within macrophages.

Systematic immunohistochemical analysis revealed the presence of *C. acnes* in 74% of video-assisted thoracic surgery specimens, 48% of transbronchial lung biopsy samples, and nearly 90% of lymph node biopsies obtained from patients in Japan and Germany (8). This high detection rate across diverse sample types and populations corroborates the potential involvement of *C. acnes* in sarcoid granuloma formation. The PAB antibody used in these analyses exhibited high specificity, showing no reactivity in granulomas associated with tuberculosis or sarcoid reactions unrelated to sarcoidosis, thereby supporting its reliability as a diagnostic tool for *C. acnes*-associated sarcoidosis. Notably, antigen detection was more frequent in immature granulomas, likely reflecting the degradation of bacterial components as granulomas mature, which may help explain variability in detection rates observed across lesions and patients.

In addition to pulmonary and lymphatic involvement, *C. acnes* has been detected in granulomatous lesions from internal organs that are typically not exposed to external microbial flora, including the myocardium (9), retina (10), epiretinal membrane (11), and central nervous system (CNS) (58, 59), as well as from sites that are more commonly colonized by commensal microbiota, such as the lungs (60–63), skin (64–69), and nasal cavity (70). These findings extend the pathologic presence of *C. acnes* beyond mucocutaneous barriers, supporting the hypothesis that this commensal bacterium systemically disseminates and contributes to granulomatous inflammation in multiple organs. Based on the detection of PAB-positive staining within granulomas, we refer to such cases as “*C. acnes*-associated sarcoidosis” (51).

Furthermore, a recent review article from Italy by Di Francesco et al. (71) cited multiple studies from our group and emphasized the systemic involvement of *C. acnes* in sarcoidosis, including pulmonary, lymphatic, and extrapulmonary organs. This international endorsement reinforces the global recognition of *C. acnes*-associated sarcoidosis.

While the PAB antibody (MBL, D372-3) is commercially available and highly specific, its detection sensitivity can vary depending on the immunostaining platform. The Leica BOND-III system provides sensitivity comparable to the original manual protocols (72). In contrast, the VENTANA BenchMark Ultra system requires protocol adjustments to remove mineral oil, which interferes with PAB binding and reduces staining reliability (12, 72).

In summary, the consistent detection of *C. acnes* antigens within granulomas across diverse patient samples and the lack of reactivity within granulomas of other diseases highlight its potential involvement in sarcoid granuloma formation. However, the detection of bacterial antigens alone does not conclusively prove causation, as *C. acnes* could still represent an opportunistic colonizer rather than a true pathogen. These findings nonetheless provide a strong foundation for exploring the contribution of this commensal bacterium to the pathogenesis of sarcoidosis and its potential as a diagnostic and therapeutic target.

### 2.3 Dual role of *C. acnes*: commensal and pathogen

The detection of *C. acnes* within sarcoid granulomas suggests that this bacterium may actively contribute to pathogenesis rather than merely persisting as a passive commensal. Given the importance of persistent antigens in granuloma formation, clarifying the dual nature of *C. acnes* as a benign commensal in health and a potential intracellular pathogen in disease is critical.

*C utibacterium acnes* is a commensal bacterium commonly found on the skin, as well as in the gastrointestinal tract and oral cavity (73). In healthy individuals, it resides in sebaceous follicles, helps maintain microbial homeostasis, and typically induces minimal immune responses. Under certain conditions, however, such as immune suppression, microbiome disruption, or prolonged intracellular persistence, *C. acnes* can adopt a pathogenic phenotype, contributing to chronic inflammation and biofilm-associated infections. In acne vulgaris, host factors and microbial imbalance promote extracellular overgrowth and inflammation. *C. acnes* also forms biofilms on implanted medical devices, enhancing antibiotic resistance and complicating treatment (74, 75).

Recent studies demonstrated that *C. acnes* can infect not only macrophages but also epithelial and stromal cells (76, 77). Once internalized, it may evade lysosomal degradation and persist within phagosomes (78). Some intracellular bacteria, including *C. acnes*, enter a dormant state, thereby establishing latent infections (79, 80). Latent *C. acnes*, similar to MTB, which also establishes latent infection, may reactivate under immunosuppression, biologic therapy, or physiologic stress. Its ability to persist and reactivate intracellularly provides a mechanistic explanation for the chronic granulomatous inflammation observed in sarcoidosis.

### 2.4 Intracellular reactivation of latent *C. acnes*

**FIGURE 5:**
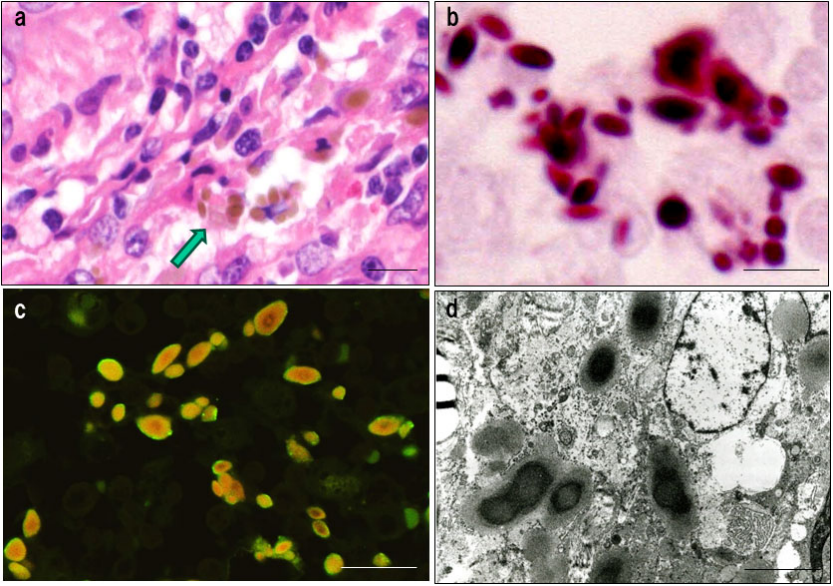
Histologic, immunohistochemical, and ultrastructural features of Hamazaki-Wesenberg (HW) bodies. This figure demonstrates key characteristics supporting the identification of HW bodies as latent intracellular *C. acnes* in an L-form state. **(a)** HE staining: HW bodies appear as yellow-brown ovoid or spindle-shaped inclusions within sinus macrophages (arrow). **(b)** Acid-fast staining: HW bodies show dark red staining, resembling acid-fast bacterial structures. **(c)** Dual immunofluorescence: HW bodies exhibit peripheral green fluorescence with the PAB antibody (membrane-bound lipoteichoic acid) and diffuse orange fluorescence with the TIG antibody (ribosome-associated trigger factor), indicating preserved bacterial integrity. **(d)** Electron microscopy: HW bodies contain an electron-dense core and lack a typical bacterial cell wall, consistent with L-form bacteria. Scale bar: 5 μm. All images are original.

Latent *C. acnes* can persist intracellularly in a cell wall-deficient form and may undergo reactivation under specific conditions, thereby initiating granulomatous inflammation. A characteristic morphologic indicator of this latent state is the Hamazaki-Wesenberg (HW) body, a spindle- or ovoid-shaped cytoplasmic inclusion found in sinus macrophages of sarcoid lymph nodes (81). These structures exhibit acid-fast staining and are strongly immunoreactive to both PAB and trigger-factor protein (TIG) antibodies, the latter of which targets a ribosome-associated trigger factor protein of *C. acnes* (Figure 5).

**FIGURE 6:**
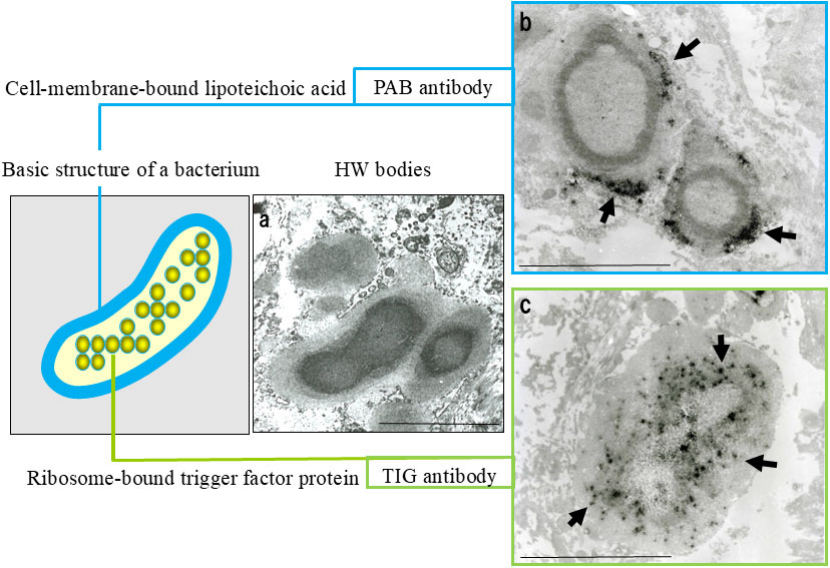
Evidence suggesting that Hamazaki-Wesenberg (HW) bodies represent cell wall-deficient *C. acnes*. Ultrastructural and immunoelectron microscopic findings supporting the identification of HW bodies as L-form *C. acnes*. **(a)** Electron microscopy: HW bodies exhibit an electron-dense core with a less dense periphery and lack a conventional bacterial cell wall. **(b)** Immunoelectron microscopy with the PAB antibody (targeting membrane-bound lipoteichoic acid) shows black signals (arrows) at the periphery, indicating bacterial membranes. **(c)** Immunoelectron microscopy with TIG antibody (recognizing ribosome-bound trigger factor) shows dispersed black signals (arrows) throughout the interior, indicating bacterial ribosomes. These features indicate that HW bodies retain the original localization of key bacterial components despite the absence of a cell wall. Scale bar: 5 μm. All images are original and previously published (8, 22, 104).

The absence of a cell wall in HW bodies may facilitate immune evasion and support long-term intracellular persistence. Immunoelectron microscopy using PAB and TIG antibodies has confirmed the structured localization of both cell membrane-bound LTA and the ribosomal trigger-factor protein within HW bodies (Figure 6), supporting their identification as L-form *C. acnes*, a latent and cell wall-deficient variant associated with chronic intracellular persistence (80).

**FIGURE 7:**
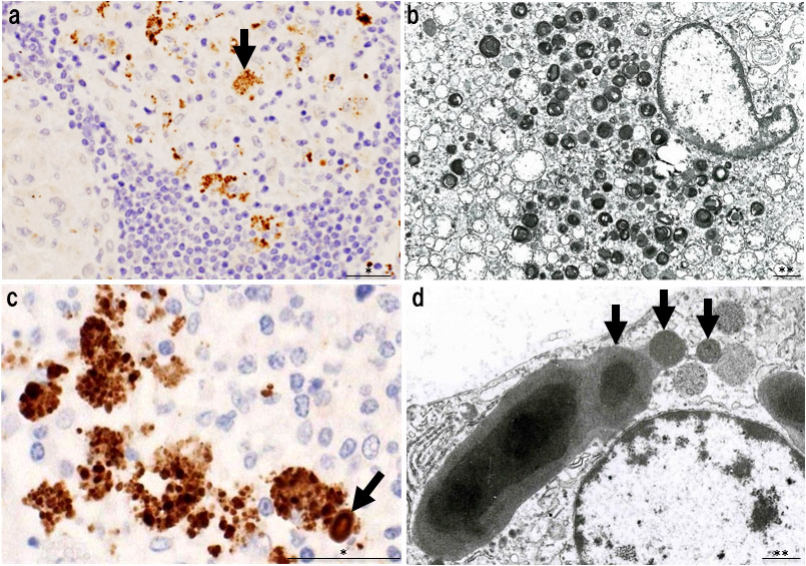
Features of intracellular *C. acnes* proliferation in sarcoid lymph nodes. Intracellular proliferation of *C. acnes* in sarcoid lymph nodes, based on *P. acnes* specific monoclonal antibody (PAB) immunohistochemistry and electron microscopy. **(a)** Immunohistochemistry with PAB antibody shows numerous positive signals (arrow) within granuloma cells, indicating intracellular accumulation of *C. acnes* antigens. **(b)** Electron microscopy reveals corresponding small, round electron-dense bodies, some with lamellar structures, suggesting partial degradation during intracellular digestion. **(c)** In paracortical regions, clusters of swollen macrophages contain dense accumulations of PAB-reactive bodies. A PAB-positive HW body is also observed (arrow), suggesting a link between intracellular proliferation and HW body formation. **(d)** Electron microscopy shows small round bodies extruding from an ovoid HW body (arrows), consistent with a budding-like proliferation process of latent *C. acnes*. These findings suggest that *C. acnes* proliferates intracellularly within macrophages and may contribute to granuloma formation in sarcoidosis. Scale bars: *50 μm, **1 μm. All images are original and previously published (22, 51).

In granulomas containing abundant PAB-positive small round bodies, some macrophages show cytoplasmic accumulation suggestive of intracellular bacterial proliferation (Figure 7). Electron microscopy has verified that these round bodies represent small, electron-dense *C. acnes* undergoing partial degradation. Clusters of enlarged macrophages in the paracortical region frequently contain numerous PAB-positive small round bodies along with occasional HW bodies. Budding-like extrusion of round bodies from HW structures suggests that HW bodies function as intracellular reservoirs capable of reactivation. This transition likely reflects a shift from latent, cell wall-deficient persistence to active replication.

Together, these findings suggest that reactivation of intracellular latent *C. acnes* may serve as an initiating event in granuloma formation. The process may involve interactions with autophagy and host immune recognition pathways, providing a mechanistic link between latent infection and the chronic granulomatous inflammation observed in sarcoidosis. This model provides a plausible explanation for histologic variation in sarcoid granulomas, reflecting different stages of bacterial activity and immune response.

### 2.5 Molecular studies of *C. acnes*

**FIGURE 8:**
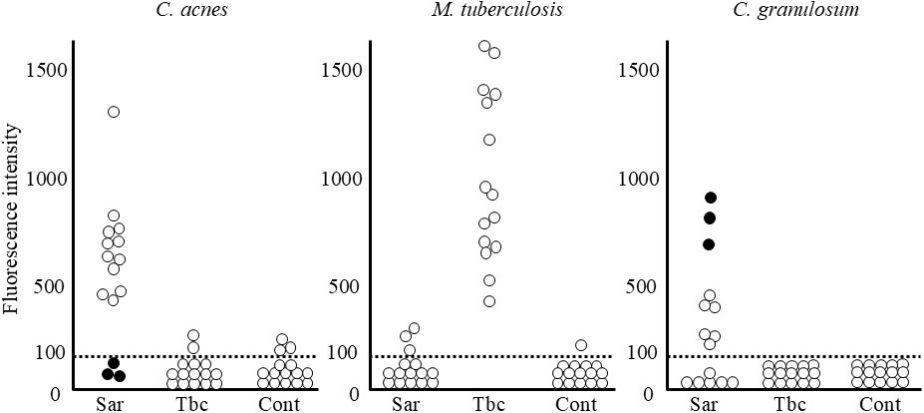
Quantitative polymerase chain reaction (PCR) analysis of bacterial DNA in lymph nodes from patients with sarcoidosis (Sar), tuberculosis (Tbc), and gastric cancer (Cont). This analysis used quantitative PCR to detect DNA from *C. acnes*, *M. tuberculosis* (MTB), and *C. granulosum* in lymph node samples. Fluorescence intensity reflects bacterial DNA quantity; the horizontal dotted line indicates the detection threshold, below which samples were considered PCR-negative. In sarcoidosis, *C. acnes* DNA was detected at levels comparable to MTB in tuberculosis, supporting its potential role in granuloma formation. Three sarcoidosis patients negative for *C. acnes* (black dots) showed high *C. granulosum* DNA levels, suggesting its possible involvement in some cases. Outside of disease-associated cases (i.e., *C. acnes* in sarcoidosis and MTB in tuberculosis), both bacteria were occasionally detected near the threshold, indicating a low-level presence consistent with latent infection. All images are original and previously published (51, 104).

Molecular techniques, particularly real-time PCR, have provided strong molecular evidence supporting the involvement of *C. acnes* in sarcoidosis (Figure 8). Quantitative PCR consistently detects significantly higher levels of *C. acnes* 16S rRNA DNA in sarcoid granulomas than in non-sarcoid controls across diverse populations, including those from Japan (6, 7), China (82), Europe (7), and North America (83). This cross-regional consistency suggests that *C. acnes* represents a globally relevant microbial agent in sarcoidosis.

Studies from Japan and China report elevated levels of *C. acnes* and *C. granulosum* DNA in sarcoid lymph nodes, suggesting the potential involvement of multiple *Cutibacterium* species. The role of *C. granulosum* remains uncertain, however, as it is not recognized by the PAB antibody. Notably, Ichikawa et al. (84) observed markedly increased *C. acnes* DNA in BAL cells from Japanese patients with sarcoidosis, whereas *C. granulosum* DNA was rarely detected in either patients or controls, supporting *C. acnes* as the predominant species associated with sarcoidosis.

Two meta-analyses further support this finding. Esteves et al. (3), analyzing 58 case-control studies, reported a strong link between *C. acnes* and sarcoidosis (OR 18.80; 95% CI: 12.62–28.01), primarily based on lymph node samples. In contrast, mycobacterial DNA showed a weaker and geographically limited association (OR 6.8), mostly confined to TB-endemic regions. Similarly, Zhou et al. (85), reviewing nine studies from Japan and China, reported a strong association (OR 19.58; 95% CI: 13.06–29.36), with *C. acnes* DNA detected in 78.4% of sarcoidosis cases compared to 21.7% of controls. These findings emphasize the strong and geographically consistent relationship between *C. acnes* and sarcoidosis, particularly in lymphatic tissue.

Yamada et al. (86) applied *in situ* hybridization with catalyzed reporter deposition and demonstrated that *C. acnes* DNA is concentrated within sarcoid granulomas, with a relatively prominent localization toward the periphery. Quantitative PCR confirmed the presence of *C. acnes* DNA in 89% of sarcoid samples, with higher copy numbers than those observed in tuberculous or control nodes. A strong correlation between *in situ* hybridization signal counts and PCR results (r = 0.86, *p* < 0.001) underscores the reliability of molecular methods in detecting *C. acnes* and supports its association with granulomatous inflammation.

**FIGURE 9:**
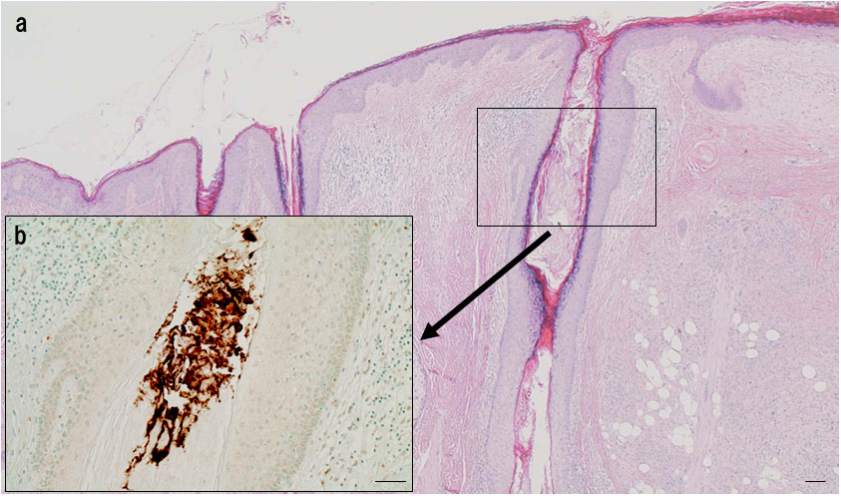
Extracellular localization of *C. acnes* in the follicular lumen of normal skin. **(a)** HE staining shows the structure of a sebaceous follicle. **(b)** Immunohistochemistry with *P. acnes* specific monoclonal antibody (PAB) antibody (targeting *C. acnes* lipoteichoic acid) reveals intense brown signals densely distributed in the follicular lumen, indicating extracellular localization. No signals are observed in follicular epithelial cells, suggesting a commensal state without intracellular invasion. Scale bar: 100 μm. All images are original.

**FIGURE 10:**
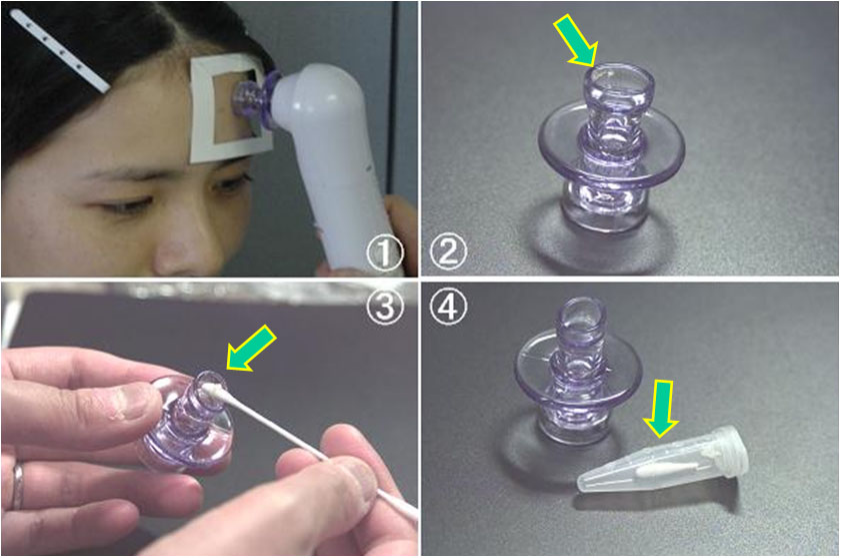
Sampling procedure for extracellular *C. acnes* from sebaceous follicles using the Harue method. This figure illustrates a non-invasive method for collecting extracellular *C. acnes* from sebaceous follicles using a pore vacuum device for molecular analysis (Harue method). (1) The device applies suction to a specific forehead area for a standardized time to collect follicular contents. (2, 3) Collected material (arrows) is transferred onto a cotton swab. (4) The swab tip is cut, placed in a sterile tube, and stored frozen until DNA is extracted for real-time PCR targeting the *C. acnes*-specific 16S rRNA gene. All photos are original and previously published (87).

**FIGURE 11:**
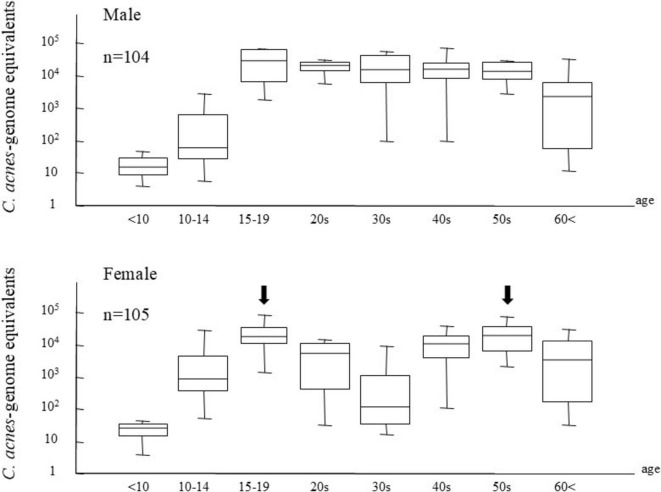
Age-related changes in extracellular *Cutibacterium acnes* load in normal sebaceous follicles. Median and range of *C. acnes* levels in sebaceous follicles are shown by age group in healthy males (upper panel, *n* = 104) and females (lower panel, *n* = 105). *C. acnes* is rarely detected before age 10, increases thereafter, and peaks at 15–19 years in both sexes. In males, levels remain stable from the 20 to 50 s with a slight decline after 60. In females, a bimodal pattern is observed with peaks at 15–19 years and again in the 50 s (arrows), paralleling the age distribution of sarcoidosis incidence. These results suggest a potential link between *C. acnes* colonization dynamics and sarcoidosis development. All images are original and previously published (87).

While *C. acnes* is a common extracellular skin commensal in sebaceous follicles (Figure 9), understanding its baseline abundance is essential for evaluating its pathogenic potential in sarcoidosis. Using a specialized follicular sampling method (Figure 10) combined with real-time PCR, its bacterial load was quantified in healthy individuals (87). The bacterial burden increased after age 10, peaking between ages 15 and 19 (Figure 11), a pattern that coincides with the typical onset age of sarcoidosis (20–29 years). In females, *C. acnes* bacterial load exhibits a bimodal distribution, with a second peak occurring in the fifth decade, paralleling the later incidence peak of sarcoidosis between ages 45 and 65 (88). It remains unclear, however, whether extracellular bacterial load accurately reflects latent intracellular infection. Nonetheless, the possibility that an increased extracellular bacterial load might correlate with a higher risk of intracellular persistence cannot be excluded.

### 2.6 Culture studies of *C. acnes*

Although molecular techniques are now widely used, bacterial culture historically provided the first evidence for the presence of viable *C. acnes* in sarcoidosis. Unlike PCR, which detects DNA fragments, culture directly demonstrates live, replicating bacteria and therefore provides more direct evidence of pathogenic relevance.

Culturing *C. acnes* is technically demanding due to its slow growth rate, strict anaerobic requirements, and the need for prolonged incubation, which often exceeds 2 weeks, to isolate viable organisms from clinical specimens. Despite these challenges, *C. acnes* is the only microorganism that has been successfully isolated from sterile lymph nodes of sarcoidosis patients in both Japan (26, 27) and the Netherlands (28). Notably, in Japan, culture positivity was significantly higher in sarcoidosis patients (78%) than in non-sarcoid controls (25%), supporting its pathogenic involvement rather than its presence due to contamination.

Ishige et al. (6) reported significantly higher *C. acnes* DNA copy numbers in sarcoid lymph nodes compared to controls using quantitative PCR, suggesting that increased bacterial load may facilitate positive culture results. In a separate study (89), *C. acnes* was cultured at low levels (< 500 CFU/g) from 56% of peripheral lung tissues and 73% of mediastinal lymph nodes in patients without sarcoidosis. These samples, obtained from individuals with lung cancer, were histologically normal, indicating that *C. acnes* can act as a commensal in some internal organs, though not consistently observed.

The observed capacity of *C. acnes* to convert from a cell wall-deficient (L-form) state to a culturable form may be associated with its potential for intracellular persistence and reactivation. Its isolation from granulomatous lesions and otherwise sterile tissues, along with its immunohistochemical detection in sarcoid granulomas and sinus macrophages of both sarcoid and non-sarcoid lymph nodes, supports the hypothesis that intracellular *C. acnes* plays an active role in the pathogenesis of sarcoidosis beyond its role as a mere cutaneous commensal.

Importantly, the culture positivity rate in sarcoid lymph nodes (78%) closely parallels the PCR detection rate (78.4%), whereas in non-sarcoid controls, culture (25%) and PCR (21.7%) produced similarly low detection rates. This concordance between methods supports the reliability of both approaches and the interpretation that viable *C. acnes* may contribute to disease pathogenesis.

Detection of *C. acnes* in non-sarcoid tissues by both culture and PCR suggests that latent intracellular infection may occur even in asymptomatic individuals, although this does not necessarily establish a direct causal relationship with disease. Furthermore, immunohistochemical and ultrastructural studies have identified cell wall-deficient forms, such as HW bodies, within sinus macrophages of lymph nodes in both sarcoid and non-sarcoid cases,

indicating that latent infection alone is insufficient to cause sarcoidosis. These observations imply that host genetic and immunological susceptibility to *C. acnes* may play a more critical role in disease pathogenesis than the mere presence of the bacterium itself.

### 2.7 Immune complex formation induced by *C. acnes*

Serologic analysis of anti-*C. acnes* LTA antibodies indicates early-life exposure, with titers rising after puberty and remaining detectable in all adults, suggesting persistent antigenic stimulation throughout life. IgG, IgA, and IgM levels, however, are not significantly different between healthy controls and sarcoidosis patients (90).

Suzuki et al. (91) identified insoluble immune complexes in sinus macrophages of sarcoid lymph nodes, while Uchida et al. (90) detected circulating immune complexes in plasma, indicating both localized and systemic humoral immune responses to *C. acnes*. Notably, immune complexes are absent within granulomas, supporting the hypothesis that granulomas result from intracellular bacterial reactivation rather than from uptake of extracellular antigen.

**FIGURE 12:**
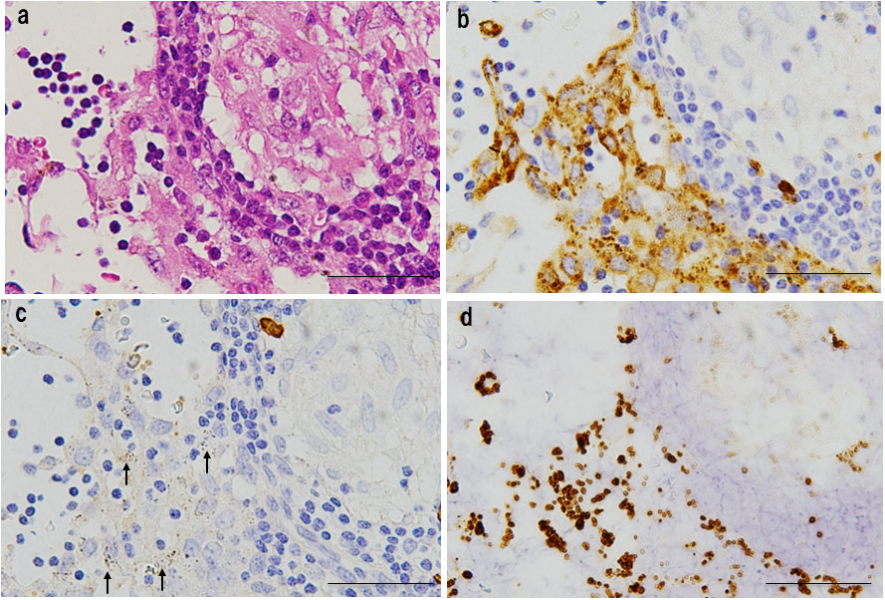
Insoluble immune complexes in the lymphatic sinuses of sarcoidosis lymph nodes. These images show an epithelioid granuloma in the paracortical region (top right) and the adjacent lymphatic sinus of a sarcoidosis lymph node. **(a)** HE staining; **(b)** IgA immunostaining; **(c)** IgG immunostaining; **(d)** *P. acnes* specific monoclonal antibody (PAB) immunostaining after trypsin treatment to unmask *C. acnes* antigens by partially digesting bound immunoglobulins. All panels depict the same region. Granular material phagocytosed by sinus macrophages is IgA-positive but IgG-negative (arrows), indicating IgA-bound immune complexes. These complexes show ring-like PAB positivity, which is consistent with *C. acnes* membrane-bound lipoteichoic acid. Additional IgM staining (data not shown) supports the presence of IgM in the complexes. These findings suggest that the complexes consist of *C. acnes* bound to IgA and IgM. Scale bar: 40 μm. All images are original and previously published (91).

In sarcoid lymph nodes, insoluble immune complexes composed of *C. acnes* LTA, immunoglobulins (primarily IgA and IgM), and complement proteins (C1q, C3c) accumulate in sinus macrophages (Figure 12). This likely reflects the attempted clearance of *C. acnes* that escaped from macrophages following intracellular proliferation. Some bacteria, however, may evade clearance and return to a latent state. The predominance of IgA and IgM suggests a mucosal or innate-type immune response, whereas IgG-associated complexes may reflect systemic immune activation. The absence of antibody binding within granulomas supports the concept that cell-mediated responses targeting intracellular *C. acnes*, rather than humoral responses, are central to granuloma formation.

Many patients exhibit elevated blood levels of *C. acnes*-derived circulating immune complexes, a condition known as antigenemia, suggesting ongoing leakage of bacterial antigens into the circulation. This may contribute to systemic immune activation. Antigenemia is a well-established feature of chronic viral infections, such as cytomegalovirus (92), in which persistent antigen leakage sustains immune stimulation. A similar mechanism may link local *C. acnes* reactivation to systemic manifestations such as fatigue in sarcoidosis (93).

Although immune complex formation facilitates the clearance of extracellular bacteria, it does not eliminate intracellular *C. acnes*. While antigenemia may contribute to systemic symptoms, it does not explain granuloma formation. Thus, the primary pathogenic mechanism in sarcoidosis appears to involve dysregulated cell-mediated immunity targeting intracellularly replicating bacteria, which explains both the absence of immune complexes in granulomas and the predominance of T cell-driven inflammation.

### 2.8 Cellular immune responses to *C. acnes*

*C utibacterium acnes* is a lifelong skin and mucosal commensal. Although most individuals develop high titers of anti-LTA antibodies, they typically lack strong cellular immune responses, likely due to Treg-mediated suppression of antigen-specific T cells that maintains immune tolerance (94, 95). Such tolerance may play a key role in preventing pathologic immune activation in response to non-pathogenic colonization.

Nakata et al. (13), Mori et al. (14) showed that alveolar lymphocytes from untreated sarcoidosis patients exhibited strong proliferation and IL-2 production in response to *C. acnes* extracts, whereas alveolar lymphocytes from non-sarcoidosis controls (including healthy individuals and lung cancer patients) did not show such responses. In contrast, peripheral blood mononuclear cells (PBMCs) from either patients or healthy individuals also failed to respond. This localized T-cell response correlates with disease activity and serum biomarkers, suggesting its involvement in granuloma formation and maintenance.

Schupp et al. (15) reported elevated levels of anti-*C. acnes* IgG and IgA antibodies in the BAL fluid of sarcoidosis patients, consistent with prior antigen exposure. BAL cells also produced TNF-α and granulocyte-macrophage colony-stimulating factor upon stimulation with heat-killed *C. acnes*, further implicating the bacterium in local immune activation.

Furusawa et al. (16) demonstrated that PBMCs from sarcoidosis patients showed increased production of Th1 cytokines (IL-2, IL-12) but reduced levels of the Th17 cytokine IL-17 in response to viable *C. acnes*, indicating a Th1-skewed immune profile that may contribute to chronic granulomatous inflammation.

Other studies have identified key bacterial antigens involved in immune dysregulation. Ebe et al. (17) showed that the *C. acnes* trigger factor, a bacterial chaperone protein, elicited variable immune responses; in some sarcoidosis patients, it induced robust PBMC proliferation and antibody production, suggesting its role in antigen-specific immune activation.

Yamamoto et al. (96), Yorozu et al. (18) demonstrated that *C. acnes* catalase, which protects against oxidative stress, promotes intracellular persistence. Sarcoidosis patients exhibit elevated IFN-γ responses to catalase. Catalase has been localized to HW bodies in sinus macrophages, and immunoelectron microscopy confirmed that HW bodies are cell-wall-deficient *C. acnes* expressing catalase and trigger factor, both of which likely promote immune evasion and chronic persistence.

Catalase and trigger factor are likely upregulated during intracellular proliferation of *C. acnes* and may serve as immunodominant antigens in sarcoidosis. Catalase enhances bacterial survival under oxidative stress, while trigger factor acts as a molecular chaperone essential for protein folding. Their presumed increased expression within host cells may facilitate antigen processing and presentation via MHC class II pathways. In individuals with impaired immune tolerance, these antigens could trigger exaggerated Th1 responses, thereby contributing to granulomatous inflammation.

Although *C. acnes* LTA is consistently detected within granulomas by immunohistochemistry, catalase and trigger factor have not been similarly identified in these lesions. A plausible explanation is that these protein antigens, unlike the more stable glycolipid structure of LTA, are rapidly degraded by lysosomal enzymes in differentiated epithelioid cells. Due to their enhanced phagolysosomal activity, these cells may eliminate intracellular proteins. This difference in antigen stability may explain the immunohistochemical absence of catalase and trigger factor within granulomas despite strong T-cell responses to these antigens in sarcoidosis patients.

Importantly, intracellular proliferation of *C. acnes* may occur even in healthy individuals without eliciting an inflammatory response. In sarcoidosis, granulomatous inflammation is thought to develop when immune tolerance is disrupted, as suggested by the presence of an exaggerated Th1 response in combination with insufficient Treg-mediated suppression. This view supports the concept that failure of immune regulation, rather than bacterial proliferation alone, may play a critical role in determining whether *C. acnes* persists as a harmless commensal or instead acts as a pathogenic trigger in sarcoidosis.

### 2.9 Animal models for *C. acnes*- associated sarcoidosis

Granuloma formation in response to *C. acnes* may occur only in genetically predisposed “high responders” capable of mounting strong cellular immune responses. This concept is supported by the Kveim reaction (56), a delayed-type hypersensitivity test that reflects antigen-specific T-cell activation. Thus, host genetic and immunologic predisposition, rather than antigen exposure alone, appear to be critical determinants of granuloma development.

Experimental autoimmune models further support this concept. In experimental autoimmune thyroiditis (EAT), subcutaneous immunization with thyroglobulin and complete Freund’s adjuvant induces thyroiditis in susceptible rat strains (e.g., DA) but not in resistant strains (e.g., PVG), despite similar antibody production (97). This dissociation between humoral and cellular immune responses parallels sarcoidosis if *C. acnes* functions as an intracellular antigen. Moreover, EAT is self-limiting in healthy rats, with remission followed by resistance to reinduction, a pattern that resembles the spontaneous remission observed in some sarcoidosis cases (98, 99).

Morris et al. (100) demonstrated that naturally existing CD4^+^CD25^+^Foxp3^+^ Tregs maintain immune tolerance in EAT; the depletion of Tregs leads to enhanced thyroiditis, highlighting their role in suppressing pathogenic effector T-cell responses. This finding is relevant to sarcoidosis, where Treg dysfunction may similarly permit granulomatous inflammation.

Murine sarcoidosis models mimic EAT: subcutaneous immunization with *C. acnes* and complete Freund’s adjuvant induces pulmonary granulomas (101–103). Minami et al. (102) found that 25%–57% of immunized mice developed lung granulomas, and *C. acnes* was cultured from normal lung tissue in 33% of mice under specific pathogen-free conditions. Antimicrobial treatment during immunization suppressed granuloma formation, indicating that persistent bacterial infection and antigen retention in the lung are required for disease induction (101, 104). This mirrors human findings, where *C. acnes* is isolated from lung and mediastinal lymph nodes in 50%–60% of adults (89), suggesting that latent infection could serve as a cryptic antigen in genetically and immunologically susceptible individuals.

Experimental findings suggest that loss of immune tolerance is a major contributor to granuloma formation in these models. Kamata et al. (105) showed that ICAM-1-deficient mice develop more severe *C. acnes*-induced pulmonary granulomas due to impaired IL-10–producing Tregs, implicating regulatory failure in the pathogenesis of experimental pulmonary sarcoidosis. Similar mechanisms have been observed in EAT, further supporting the critical role of defective Treg-mediated suppression (100).

Additional evidence for the involvement of *C. acnes* in immune dysregulation is its ability to induce IL-17A–mediated immune responses. Although IL-17A plays a key role in protecting mucosal surfaces, excessive or uncontrolled IL-17A activity promotes chronic inflammation in both EAT and *C. acnes*-induced sarcoidosis models (106, 107). In this context, latent *C. acnes* infection may function similarly to a self-antigen. When persistent antigenic stimulation transiently disrupts immune tolerance mechanisms by intracellular latent *C. acnes* reactivation, this can lead to pathologic immune activation. This concept further strengthens the hypothesis that *C. acnes* acts as an endogenous antigen driving granulomatous inflammation in sarcoidosis.

## 3 Pathogenesis and immunologic mechanisms of sarcoidosis

Sarcoidosis is characterized by dysregulated cell-mediated immunity and the formation of non-caseating granulomas. Its pathogenesis likely involves persistent antigenic stimulation, defective immune regulation, and genetic predisposition (108). The interplay among Th1/Th17-dominant responses, impaired Treg-mediated tolerance, and microbial persistence, particularly of *C. acnes*, is thought to be central to disease progression.

### 3.1 Autoimmune characteristics in sarcoidosis

Although sarcoidosis is a granulomatous inflammatory disease, it shares key immunologic features with organ-specific autoimmune disorders (2). One such shared feature is the persistent activation of Th1 and Th17 cells, which in sarcoidosis occurs within granulomas and leads to elevated production of IFN-γ, IL-17, and TNF-α, further amplifying macrophage activation and sustaining the granulomatous response (109, 110). Unlike systemic autoimmune diseases such as lupus or rheumatoid arthritis, which involve widespread autoantibody production, sarcoidosis exhibits localized immune dysregulation. Although some patients show elevated antinuclear antibodies, broad-spectrum autoantibody responses are uncommon, resembling patterns observed in tissue-specific autoimmune conditions (111, 112).

In disorders like type 1 diabetes, multiple sclerosis, and autoimmune thyroiditis, Treg dysfunction permits targeted inflammatory responses. Similarly, sarcoidosis displays impaired Treg function localized within granulomatous lesions, while systemic Treg levels may remain normal or even elevated (31, 113). This compartmentalized failure of immune regulation contributes to persistent granulomatous inflammation, paralleling organ-specific autoimmune diseases.

Persistent intracellular *C. acnes* antigens frequently detected within granulomas, may act as a chronic trigger for Th1/Th17 inflammation, perpetuating granulomatous inflammation similarly to self-antigens in autoimmune diseases. Th17.1 cells, a subset that produces both IL-17 and IFN-γ, are implicated in chronic granulomatous immune responses. Ramstein et al. (110) found increased Th17.1 cells in sarcoidosis patients, correlating with disease progression. Zhang et al. (32) reported that an imbalance between Th17.1 and Treg was associated with fibrosis severity. Th17.1 cells may evade Treg-mediated suppression via IL-23–STAT3 signaling, thereby promoting their persistence and expansion, a mechanism that has also been implicated in organ-specific autoimmune diseases (114, 115).

These findings support the notion that sarcoidosis involves antigen-specific immune responses, with *C. acnes* antigens potentially acting in a manner similar to self-antigens. Variability in Treg function, antigen persistence, and host genetics likely underlies the diverse clinical presentations of sarcoidosis (31, 113). Persistent *C. acnes* stimulation may help explain the contrast between spontaneous remission in some patients and chronic fibrotic progression in others, similar to autoimmune thyroiditis (116). Thus, both antigen persistence and immune regulatory failure likely drive the disease heterogeneity observed in sarcoidosis.

### 3.2 The role of autophagy in sarcoidosis

The pathogenic hypothesis of *C. acnes* in sarcoidosis posits that latent intracellular infection within macrophages can reactivate and proliferate, but may lead to granulomatous inflammation only in individuals with impaired Treg-mediated immune tolerance. Reactivation of intracellular *C. acnes* may initially trigger autophagy, a host defense mechanism that facilitates bacterial clearance through the fusion of autophagosomes with lysosomes (117).

In healthy individuals, autophagy contributes to immune homeostasis by promoting the clearance of intracellular bacteria, thereby potentially preventing granuloma formation (118). Although *C. acnes* is generally regarded as a commensal organism, its intracellular persistence necessitates immune surveillance. Continuous immunologic interaction may shape host immunity, promoting a balanced state that suppresses excessive inflammation and maintains tissue integrity (19).

The recruitment of autophagy to regulate intracellular microbes represents a conserved defense strategy. This mechanism closely parallels the host response to latent MTB, a well-characterized model of latent infection. In both latent *C. acnes* and MTB infection, intracellular persistence can be asymptomatic for extended periods; however, reactivation may occur under conditions of immune dysfunction. In MTB infection, autophagy serves to constrain bacterial proliferation, whereas inhibition of this pathway allows intracellular survival and dissemination (119).

In sarcoidosis, reactivated *C. acnes* may induce autophagy in macrophages as an initial protective response; however, impaired Treg function may dysregulate this process, leading to enhanced antigen presentation and Th1/Th17 responses (120). Autophagy in Tregs themselves has also been reported to play dual roles, either supporting immune tolerance or promoting inflammation depending on the context (121). Persistent activation of mechanistic target of rapamycin complex 1 (mTORC1), which is commonly observed in sarcoid granulomas, further impairs autophagy. mTORC1 not only inhibits autophagic flux but also promotes glycolysis-driven metabolic reprogramming, leading to proinflammatory cytokine production, macrophage hypertrophy, and sustained granuloma formation (122–125).

Thus, the *C. acnes*-associated model of sarcoidosis highlights the dual nature of autophagy, which serves as a protective mechanism under homeostatic conditions but contributes to pathogenesis when dysregulated by Treg dysfunction and mTORC1 activation. Understanding the dynamic interplay among *C. acnes* reactivation, autophagy, and immune regulation is critical not only for elucidating sarcoidosis pathogenesis but also for informing the development of targeted therapeutic strategies (126).

### 3.3 Tolerance induction to commensal bacteria

The establishment of immune tolerance to commensal bacteria during early life is critical for preventing maladaptive immune responses later in life. In sarcoidosis, Treg dysfunction, particularly in response to *C. acnes*, may stem from insufficient microbial exposure during early immune development. Factors such as the timing of the microbial encounter, host genetics, and epigenetic influences shape long-term immune regulation (127).

Fetal and neonatal exposure to self-antigens and commensal microbes such as *C. acnes* is critical for proper immune programming. Inadequate exposure to antigens during this developmental window can impair tolerance induction and promote maladaptive immune responses (128). For example, fetal rats deprived of thyroid antigen exposure developed autoimmune thyroiditis in adulthood following syngeneic thyroid grafting, demonstrating that the absence of specific antigen exposure during fetal life can impair the establishment of self-tolerance (129). This is supported by findings that the developmental expression of autoimmune target antigens is essential for establishing immune tolerance during organogenesis (130). Likewise, early microbial contact promotes antigen-specific Treg differentiation, supporting immune homeostasis and limiting inflammation (127, 128, 131).

The “Hygiene Hypothesis” further proposes that reduced microbial exposure in early life impairs Treg development, facilitating immune dysregulation (132). Excessive hygiene and early antibiotic use reduce microbial diversity, thereby weakening antigen presentation and Treg induction (133). Limited microbial diversity also compromises dendritic cell activation and the presentation of Treg-supportive antigens (131, 134).

Altered microbial exposure patterns, characterized by insufficient exposure during early life but increased exposure later, may preferentially enhance effector T cell activation rather than tolerance (132). Factors such as Cesarean delivery, dietary changes, and medication use can disrupt microbiota composition (127). This disruption may impair systemic Treg development and disturb mucosal-systemic immune signaling, contributing to immune imbalance.

Taken together, this framework may also help explain the bimodal age distribution of sarcoidosis, particularly among women (88). Disease onset during early adulthood (20–30 s) may be driven by heightened Th1/Th17 responses following broad microbial exposure in individuals with suboptimal tolerance mechanisms (31, 135). In contrast, disease onset after age 45 may reflect an age-related decline in the Treg number or function. In this model, early microbial exposure not only calibrates effector responses but is also crucial for establishing a stable Treg compartment. In highly sanitized environments, reduced exposure may compromise Treg maturation, leading to immune dysregulation that manifests clinically in middle-aged or older adults.

Host genetic factors further modulate immune tolerance. Sarcoidosis-associated HLA class II alleles, including HLA-DRB1*03:01 and HLA-DRB1*15:01, may limit the presentation of *C. acnes* antigens, favoring Th1/Th17 polarization (120). Prenatal exposure, including maternal infection or inflammation, may further impair Treg differentiation via epigenetic mechanisms (132). Moreover, animal studies demonstrate that neonatal antigen exposure induces durable Treg-mediated tolerance, whereas delayed exposure favors effector T cell responses (128). Hence, these genetic and environmental factors together may explain why only a subset of individuals exposed to *C. acnes* develop sarcoidosis, reflecting individual differences in early antigen exposure, antigen-specific Treg differentiation, and host genetic background (130, 132).

### 3.4 Tolerance failure to *C. acnes*

A breakdown in immune tolerance to *C. acnes* is hypothesized to play a central role in the pathogenesis of sarcoidosis by promoting granuloma formation and persistence through sustained Th1/Th17-driven inflammation. This breakdown may result from defective Treg function, insufficient induction of antigen-specific Tregs, or dysregulated antigen presentation by antigen-presenting cells.

In healthy individuals, Tregs suppress excessive Th1/Th17 responses to self-antigens and commensal microbes, thereby maintaining immune homeostasis and preventing pathologic inflammation (136). In sarcoidosis, both decreased Treg numbers and impaired Treg function may lead to inadequate control of inflammatory responses (32).

Mechanistically, Treg dysfunction in sarcoidosis may involve reduced FoxP3 expression, diminished suppressive activity, and a shift toward proinflammatory cytokine profiles (137). This dysfunction can enhance Th1/Th17 inflammation and contribute to granuloma formation. Proinflammatory cytokines such as IL-6 and IL-1β further inhibit Treg activity, favoring Th1/Th17 polarization (138). FoxP3^+^ Tregs within sarcoid granulomas often fail to suppress macrophage activation and cytokine production, contributing to persistent inflammation and granuloma maintenance (122).

Dendritic cells (DCs) in sarcoidosis also exhibit enhanced antigen presentation and a proinflammatory phenotype, further amplifying the Th1/Th17 responses (139). Although intracellular *C. acnes* can persist and even proliferate in healthy individuals without causing disease, granuloma formation in sarcoidosis likely reflects a failure to maintain antigen-specific tolerance. This failure leads to pathogenic immune responses against a normally harmless commensal.

In sarcoidosis, persistent immune activation may be sustained by the reactivation and proliferation of latent intracellular *C. acnes*, perpetuating Th1/Th17 inflammation even in the absence of overt infection. A similar mechanism is observed in secondary tuberculosis, in which latent MTB reactivates and induces granulomatous responses (140).

Genetic susceptibility further modulates disease course. HLA-DRB1*03:01 is associated with acute, self-limiting sarcoidosis (e.g., Löfgren syndrome), characterized by transient Th1/Th17 activation and spontaneous resolution, whereas HLA-DRB1*15:01 is linked to chronic, progressive disease, marked by persistent Th1/Th17 responses and immune tolerance failure (35, 141). These alleles may influence antigen processing and T cell activation, increasing susceptibility to exaggerated responses to *C. acnes*.

Thus, sarcoidosis may be better conceptualized as a disease of commensal-specific immune tolerance failure. Unlike classical autoimmunity that target self-antigens, sarcoidosis involves chronic immune activation against a commensal microbe, driven by impaired Treg regulation and persistent antigenic stimulation.

### 3.5 Hypothetical etiology of sarcoidosis

**FIGURE 13:**
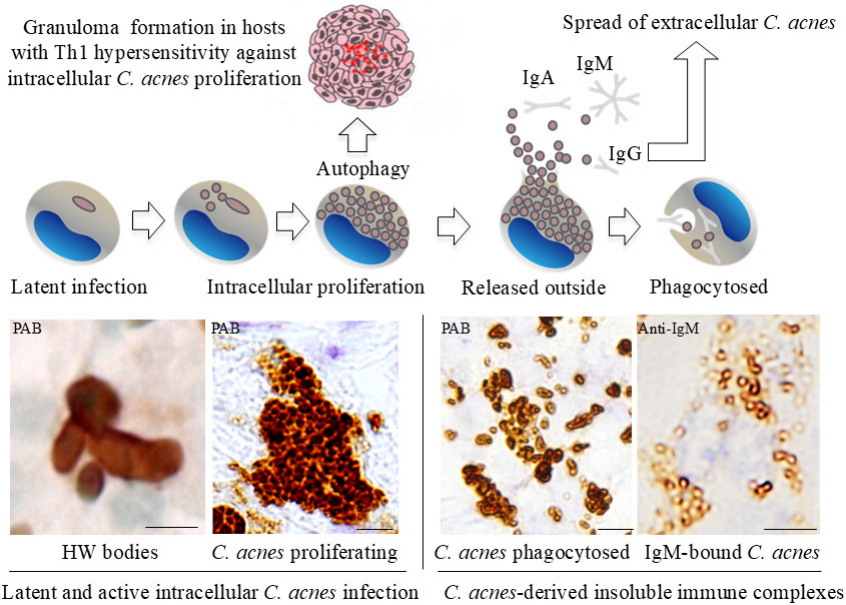
Proposed mechanism of granuloma formation driven by intracellular proliferation of latent *C. acnes*. This schematic illustrates the progression of *C. acnes* infection within macrophages in Th1-hypersensitive hosts. *C. acnes* persists intracellularly in a latent state, followed by intracellular proliferation that induces autophagy, which in susceptible individuals may trigger granuloma formation. Upon release, bacteria are opsonized by IgA, IgM, and IgG, then phagocytosed as immune complexes. These complexes may harbor viable bacteria until degraded, potentially enabling systemic dissemination and granuloma formation in multiple organs. The lower panel presents immunohistochemical findings supporting this model: (Left) HW bodies representing cell wall-deficient *C. acnes* detected by PAB antibody (latent infection). (Middle left) Intracellularly proliferating *C. acnes* strongly stained by PAB (active infection). (Middle right) Phagocytosed *C. acnes* forming insoluble immune complexes. (Right) IgM-bound *C. acnes* immune complexes stained with anti-IgM antibody. These findings support a model in which intracellular proliferation promotes granuloma formation. In this model, immune complexes may also facilitate bacterial dissemination. Scale bars: 50 μm. All images are original and previously published (22, 51).

**FIGURE 14:**
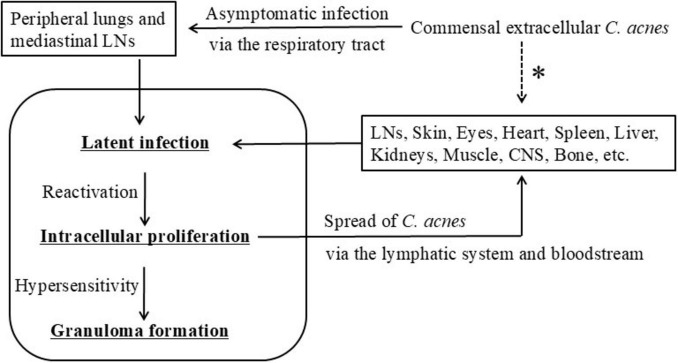
Hypothetical model of *C. acnes*-associated systemic sarcoidosis. This schematic illustrates a proposed progression of *C. acnes*-driven sarcoidosis. Commensal *C. acnes* from the skin or upper airways enters the peripheral lungs and mediastinal lymph nodes via the respiratory tract, leading to asymptomatic intracellular infection. The bacteria establish latent infection within macrophages, followed by possible reactivation and intracellular proliferation. Granulomas are hypothesized to form in individuals with impaired Treg-mediated tolerance, resulting in Th1/Th17 hypersensitivity. During this process, *C. acnes* may spread extracellularly via lymphatic or hematogenous routes to systemic organs. Dissemination of viable bacteria is presumed to occur prior to phagocytosis and intracellular digestion. The asterisk (*) indicates possible direct entry of extracellular *C. acnes* from commensal sites into systemic organs, including the skin, digestive tract, and urogenital tract. All images are original and previously published (22, 51, 104).

Building on previous studies, we propose a unified hypothesis that implicates *C. acnes* as a central microbial factor in the pathogenesis of sarcoidosis, integrating histologic, microbiologic, immunologic, and experimental evidence (Figures 13, 14) (22, 51, 104).

As a lipophilic, anaerobic skin commensal, extracellular *C. acnes* residing on the skin, nasal cavity, or oropharyngeal mucosa may enter the lower respiratory tract, likely via inhalation of desquamated epithelial debris, and subsequently be phagocytosed by alveolar or lymph node macrophages. In certain situations, *C. acnes* survives intracellular degradation and establishes latent infection in a dormant, cell wall-deficient L-form state. These L-forms evade immune detection and resist antibiotics targeting cell wall synthesis. This latency may persist asymptomatically for years under immune equilibrium. Such persistence is not limited to sarcoidosis but also occurring in healthy individuals. In this context, latent intracellular *C. acnes* may provide a low-level systemic immune stimulus that contributes to immune surveillance or homeostasis, without provoking overt inflammation.

Environmental or host-derived stressors—including fatigue, infections, immunomodulatory therapies, or redox imbalance—may disrupt this equilibrium and trigger reactivation of latent *C. acnes*. Reactivated bacteria proliferate intracellularly and stimulate host autophagy, which functions in bacterial containment and degradation. In individuals with impaired Treg-mediated tolerance to *C. acnes*, this reactivation may elicit a Th1/Th17-polarized hypersensitivity response, leading to effector T cell activation, macrophage recruitment, and granuloma formation.

Following reactivation, some replicating bacteria may exceed the phagolysosomal degradation capacity of macrophages, resulting in host cell lysis and *C. acnes* escape into the extracellular space. If these extracellular *C. acnes* are not fully phagocytosed by sinus macrophages within the draining lymph nodes, they may disseminate via efferent lymphatic vessels and enter the bloodstream, seeding extrapulmonary sites such as the heart, skin, eyes, or CNS.

At these secondary sites, *C. acnes* may reestablish latency within vascular endothelial cells or tissue-resident macrophages. Reactivation at individual sites may then induce local granulomatous inflammation, contributing to the systemic and multi-organ manifestations of sarcoidosis. In many cases, granulomatous lesions arise simultaneously in multiple organs, suggesting that endogenous reactivation of latent *C. acnes* can occur in a coordinated or near-synchronous manner. This pattern may reflect systemic immune dysregulation or shared triggering factors that promote widespread bacterial reactivation.

We hypothesize that sarcoid granuloma formation requires the convergence of three key pathogenic conditions: (1) intracellular latent infection by cell wall-deficient *C. acnes*; (2) environmental or endogenous triggers promoting bacterial reactivation; and (3) immune tolerance failure, leading to a Th1/Th17-polarized hypersensitivity response. Together, these factors drive sustained antigenic stimulation, immune dysregulation, and granulomatous inflammation.

Thus, sarcoidosis may be conceptualized as an endogenous hypersensitivity infection, wherein a normally benign commensal microbe acquires pathogenic potential through intracellular persistence, immune evasion, and host susceptibility. This model may reconcile the dual features of infection and autoimmunity observed in sarcoidosis and provide a conceptual foundation for future diagnostic and therapeutic strategies (22, 104).

### 3.6 Disease mechanism of sarcoidosis

Bilateral hilar lymphadenopathy, a hallmark of pulmonary sarcoidosis, may reflect an underlying immune response triggered by the intracellular proliferation of latent *C. acnes* within macrophages. Inhaled *C. acnes*, originating from the skin or upper airway mucosa, may reach the lower respiratory tract and be taken up by alveolar macrophages. In the presence of specific antibodies, immune complexes may enhance bacterial opsonization and intracellular uptake.

While most bacteria are eliminated, a small fraction may persist intracellularly and migrate to regional lymphatics without provoking inflammation. These bacteria may accumulate in the sinus regions of hilar and mediastinal lymph nodes, establishing a state of asymptomatic latency. Unlike tuberculosis, no primary pulmonary lesions or Ghon complexes are formed, and the infection remains immunologically silent for extended periods.

Reactivation of latent *C. acnes*, whether systemic or localized, can induce intracellular proliferation and activate macrophages within bilateral lymph nodes. Histologically, this is reflected by migration of activated macrophages from the lymphatic sinus to the paracortical T-cell zone, where granulomas begin to form. This pattern of lymph node involvement explains the symmetrical enlargement seen in stage I sarcoidosis.

A characteristic feature of pulmonary sarcoidosis is that granulomatous lesions are not randomly distributed within the alveolar spaces but are instead localized along the bronchovascular bundles, interlobular septa, and subpleural regions, which are all anatomic sites rich in lymphatic drainage (142). This suggests that latent *C. acnes*, after being phagocytosed in the alveoli, is transported via lymphatic flow into the pulmonary interstitium. There it accumulates and, upon reactivation, induces localized granulomatous inflammation. The frequent absence of granulomas within the alveolar lumen further supports the concept that sarcoid granulomas arise not from direct alveolar colonization, but from lymphatic trafficking and interstitial immune responses.

Although pulmonary involvement is most common in sarcoidosis, extrapulmonary manifestations can arise independently and exhibit diverse organ tropism (143). These lesions may occur even without active thoracic disease, suggesting that *C. acnes* can establish latent infection in extrapulmonary tissues through multiple routes of dissemination and tissue-specific adaptation. Disseminated *C. acnes* may transiently colonize vascular endothelial cells or tissue-resident macrophages in extrapulmonary sites. The efficiency of bacterial reseeding and subsequent granuloma formation likely depends on local tissue factors such as epithelial barrier integrity, immune cell composition, and organ-specific vascular architecture.

These mechanisms may explain extrapulmonary sarcoidosis without active thoracic disease. Isolated involvement of extrapulmonary organs such as the heart (144), skin (145), eyes (146), or CNS (147) may result from hematogenous dissemination of *C. acnes* originating from prior subclinical pulmonary infection or from other commensal reservoirs. These include the skin, oral cavity, gastrointestinal tract, or urogenital tract. Subsequent reactivation of latent bacteria in these extrapulmonary sites could then give rise to isolated organ-specific disease.

In scar- or tattoo-associated sarcoidosis, disruption of the epithelial barrier may permit direct cutaneous entry of *C. acnes*, leading to latent infection and subsequent granuloma formation upon reactivation in susceptible individuals (148, 149). Given that *C. acnes* is a commensal in multiple epithelial niches, direct mucosal translocation or retrograde ductal migration may also contribute to latent infection in organs such as the ocular adnexa, salivary glands, oropharyngeal and gastric mucosa, or urogenital tissues.

Rather than following a uniform pathophysiologic sequence, sarcoid lesions across different organs may arise independently, shaped by prior microbial exposure and the site-specific immune context. This model underscores the anatomic and immunologic heterogeneity of sarcoidosis while anchoring its pathogenesis to a common microbial antigenic trigger.

### 3.7 Natural history and progression of sarcoidosis

Sarcoidosis exhibits a highly variable clinical course, ranging from spontaneous remission to chronic progressive disease with organ dysfunction. Approximately two-thirds of patients experience self-limiting disease, while one-third develop persistent granulomatous inflammation and fibrosis (150). This variability reflects complex interactions among immune responses, persistent antigenic stimulation, and genetic predisposition (35, 151).

Spontaneous remission is thought to result from the restoration of Treg-mediated immune regulation, which suppresses Th1/Th17 activation and reestablishes immune homeostasis (152). Several studies emphasize the importance of Treg survival and suppressive function in achieving disease resolution (152, 153).

In contrast, chronic progression is characterized by sustained antigenic stimulation, immune dysregulation, and fibrotic remodeling (108). Inadequate clearance of intracellular *C. acnes* may perpetuate immune activation, leading to persistent Th1/Th17 responses and chronic granulomatous inflammation. Treg dysfunction contributes to ongoing inflammation, with persistent impairment associated with chronic disease (153, 154).

This clinical heterogeneity parallels findings in autoimmune disease models. In induced models such as EAT, immune regulation is generally intact, leading to transient inflammation driven by experimentally maintained antigenic sensitization (98, 99). In contrast, spontaneous models such as NOD mice (autoimmune diabetes) or MRL/lpr mice (lupus-like disease) demonstrate intrinsic tolerance failure and sustained inflammatory activity, which more closely resembles chronic sarcoidosis (155). These findings suggest that chronic sarcoidosis shares greater similarity with spontaneous autoimmunity than with transient, antigen-driven inflammation.

Unlike central tolerance, which is thymus-dependent, sarcoidosis appears to involve peripheral tolerance failure. In particular, impaired induction or function of Tregs specific to commensal antigens such as *C. acnes* may underlie this loss of tolerance. This underscores the role of peripheral immune regulation in maintaining tolerance to non-pathogenic stimuli (127).

Progressive fibrosis, particularly in the lungs, may result in irreversible architectural remodeling and respiratory insufficiency. TGF-β signaling and extracellular matrix accumulation are central to fibrotic progression, and deficient Treg-mediated control may further potentiate TGF-β activity (155, 156). Early intervention is critical, and potential therapeutic strategies include TGF-β inhibition, Treg-targeted therapies, and optimized immunomodulatory treatment regimens (157).

## 4 Treatment strategies for sarcoidosis

Although spontaneous remission occurs in many cases, the risk of chronic progression and fibrosis necessitates the development of more targeted and durable treatment strategies. Conventional therapies, including corticosteroids and immunosuppressants, are effective at controlling inflammation but are often associated with significant adverse effects and may not sufficiently address underlying immune dysregulation or microbial persistence (158).

Emerging insights into the roles of intracellular *C. acnes* and Treg dysfunction in sarcoidosis pathogenesis have highlighted the need for innovative therapeutic approaches that simultaneously target persistent antigenic stimulation and immune imbalance. These approaches aim not only to suppress inflammation but also to mitigate the immunostimulatory effects of reactivated intracellular pathogens.

A dual-modality strategy that combines antimicrobial and immunomodulatory agents may be particularly promising for patients with refractory or progressive disease. Intracellularly active antimicrobials could help control the proliferation of *C. acnes*, while immune-directed therapies may restore the functional balance between *C. acnes*-specific Th1/Th17 cells and Tregs. Together, this integrated approach holds the potential to reduce chronic inflammation and prevent fibrotic remodeling.

To support such precision-based interventions, future research should prioritize the identification of microbial and immunologic biomarkers that allow for patient stratification according to dominant pathogenic drivers. Integrating *C. acnes* detection with immune profiling may enable the development of individualized, mechanism-based treatment strategies for *C. acnes*-associated sarcoidosis.

### 4.1 Current standard of care

The primary goals of sarcoidosis treatment are to reduce inflammation, preserve organ function, and improve quality of life. Corticosteroids remain the first-line therapy when pharmacologic intervention is required, with prednisolone typically initiated at 20–40 mg/day for approximately 6 months, followed by tapering based on disease activity (159). The SARCORT trial demonstrated that 20 mg/day is as effective as 40 mg/day, supporting lower initial doses to minimize adverse effects.

Because prolonged corticosteroid use is associated with serious complications—including osteoporosis, diabetes, hypertension, and weight gain—steroid-sparing agents are often employed. These include methotrexate, azathioprine, mycophenolate mofetil, hydroxychloroquine, and leflunomide (160). Methotrexate is particularly effective for pulmonary sarcoidosis (161), while azathioprine and mycophenolate are more frequently used for extrapulmonary disease (162). These agents reduce corticosteroid dependence by inhibiting lymphocyte proliferation (54, 163).

For refractory cases, biologics targeting TNF-α (e.g., infliximab, adalimumab) have demonstrated clinical benefit. Infliximab has shown efficacy in pulmonary and neurologic sarcoidosis (164, 165), while adalimumab may be particularly beneficial in neurosarcoidosis (166, 167). However, head-to-head trials are lacking, and treatment selection should be individualized based on organ involvement and patient-specific factors. These agents carry risks of serious infections (e.g., tuberculosis, fungal infections, sepsis) and paradoxical granulomatous inflammation (168). Screening for latent infections, particularly tuberculosis, is essential before initiating biologics, and regular monitoring during therapy is recommended (169).

Despite the efficacy of these therapies in controlling inflammation, they do not directly address underlying disease mechanisms, including immune dysregulation and microbial persistence such as that caused by *C. acnes*. This highlights the need for pathogen-specific strategies that target the root cause of the persistent inflammation. Approaches that aim to eradicate intracellular *C. acnes* through antimicrobial therapies may promote sustained remission. Additionally, immunologic interventions that restore immune tolerance may reduce the long-term reliance on broad immunosuppressive therapy.

### 4.2 Antimicrobial therapy targeting *C. acnes*

Growing evidence implicating *C. acnes* in sarcoidosis has prompted the consideration of antimicrobial therapy as an adjunctive treatment (29, 170). Recent reviews, including that by Kraaijvanger and Veltkamp (29), have proposed reframing *C. acnes*-driven sarcoidosis as a “treatable trait,” emphasizing its role as both a disease-associated antigen and a therapeutic target, and highlighting the potential of biomarker-guided, pathogen-targeted interventions.

Tetracycline-class antibiotics, such as minocycline and doxycycline, are promising due to their combined antibacterial and immunomodulatory properties. By inhibiting bacterial protein synthesis at the 30S ribosomal subunit, they may suppress *C. acnes*-induced immune activation. These agents appear to target metabolically active intracellular *C. acnes*, but their efficacy against latent, cell wall-deficient forms remains limited. Prolonged tetracycline therapy has been associated with clinical improvement in cutaneous sarcoidosis (171–173).

Macrolides (e.g., clarithromycin, azithromycin) also inhibit protein synthesis at the 50S ribosomal subunit and exert anti-inflammatory effects by reducing cytokine production (174). Like tetracyclines, these agents have shown benefit in small studies, although well-designed randomized controlled trials are still needed (175, 176).

Two clinical trials are currently evaluating antimicrobial strategies for *C. acnes*-associated sarcoidosis. The J-ACNES trial in Japan (170) combines clarithromycin and doxycycline with corticosteroids in cardiac sarcoidosis. The PHENOSAR study in the Netherlands (29) investigates azithromycin and doxycycline in patients with *C. acnes*-positive granulomas. While results are pending, these trials may provide critical evidence supporting pathogen-directed therapy.

Although antibiotic monotherapy is unlikely to induce full remission, combined use with immunomodulatory agents may yield synergistic effects, particularly in patients with confirmed *C. acnes* involvement. This dual-targeted strategy may suppress replicating intracellular bacteria and mitigate chronic inflammation. Microbial diagnostics and immune profiling will be essential for identifying appropriate candidates.

Clarifying whether clinical improvement reflects immunosuppressive activity or pathogen eradication will be crucial for optimizing targeted therapy and guiding treatment decisions. Given that not all patients achieve sustained remission with pathogen-directed therapies alone, direct targeting of immunologic dysregulation has emerged as an essential complementary strategy.

### 4.3 Strategies for restoring immune tolerance in sarcoidosis

Enhancing antigen-specific Tregs may represent a potential strategy to restore immune tolerance in sarcoidosis and possibly mitigate granulomatous inflammation. Although *C. acnes*-specific Tregs have not been directly studied in sarcoidosis, they are likely to be relevant given the central role of *C. acnes* in disease pathogenesis. Emerging evidence indicates that peripheral induction of Tregs against microbial antigens is achievable in adults, particularly at mucosal sites (177, 178).

Antigen-specific Treg induction has shown efficacy in other immune-mediated diseases, such as type 1 diabetes and multiple sclerosis, by selectively suppressing pathogenic Th1/Th17 responses while preserving global immune function. In type 1 diabetes, self-antigen-specific Tregs improve glycemic control and slow disease progression (179); in multiple sclerosis, myelin-specific Tregs reduce inflammatory activity and disease severity (180). Immune checkpoint molecules such as CTLA-4 and PD-1 play essential roles in Treg function and tolerance induction, as demonstrated in various autoimmune models (181, 182). Their relevance in *C. acnes*-driven sarcoidosis, however, remains to be established.

Several tolerance-inducing approaches, including oral, nasal, or subcutaneous antigen delivery; *ex vivo* expansion or engineering of antigen-specific Tregs; low-dose IL-2 administration; and tolerogenic DC therapy, may be applicable to *C. acnes*-associated sarcoidosis, although their clinical utility has not yet been evaluated (183). Tolerogenic DCs, for example, promote Treg induction by presenting antigens in a non-inflammatory context (184).

In sarcoidosis, breakdown of tolerance to *C. acnes* likely contributes to persistent Th1/Th17 activation and granuloma maintenance. Reactivation of latent *C. acnes* under conditions of impaired regulatory control may sustain chronic inflammation. Induction of *C. acnes*-specific Tregs, such as through tolerogenic DCs (185), could restore immune homeostasis and reduce granulomatous pathology. To enable clinical translation, it will be essential to identify immunodominant *C. acnes* epitopes, such as catalase and trigger factor, and to address key challenges related to the manufacturing, scalability, and standardization of Treg-based therapies.

Unlike classical autoimmune diseases targeting self-antigens, *C. acnes*-associated sarcoidosis involves a commensal-derived antigen, raising distinct immunologic and therapeutic considerations. This distinction highlights the need for precision therapeutics approaches that integrate microbial diagnostics with immune profiling. While preclinical models are currently lacking, parallels with other antigen-driven inflammatory conditions support the feasibility of this approach. Future research should focus on developing experimental systems and validating *C. acnes*-specific Treg function in sarcoidosis patients.

### 4.4 Sarcoidosis-like reactions and latent microbial reactivation

Drug-induced sarcoidosis-like reactions (DISRs), also referred to as sarcoidosis-like reactions, have been reported in association with various immunomodulatory therapies, including TNF-α inhibitors, immune checkpoint inhibitors, IFNs, and antiretroviral therapy (186). These reactions are characterized by granulomatous inflammation that mimics sarcoidosis but typically resolves upon discontinuation of the causative agent, distinguishing them from idiopathic sarcoidosis. Differentiation can be challenging, however, when histopathologic findings are similar.

Many agents associated with DISRs are also linked to an increased risk of tuberculosis, suggesting a shared mechanism involving the reactivation of latent pathogens (187, 188). Given that both sarcoidosis and secondary tuberculosis are granulomatous disorders potentially triggered by latent pathogens, it is hypothesized that DISRs and drug-induced tuberculosis may result from reactivation of latent organisms such as *C. acnes* or MTB in susceptible individuals (22).

TNF-α plays a critical role in controlling intracellular infections. Its inhibition can impair macrophage function, permitting reactivation of latent MTB (189). TNF-α also contributes to immune homeostasis, and its blockade may promote type I IFN or Th17-skewed immune responses, thereby facilitating paradoxical immune-mediated conditions such as psoriasis or DISRs (168). The presence of *C. acnes* has been confirmed by PAB immunostaining within granulomas in a DISR case that developed during anti-TNF-α therapy (61).

Immune checkpoint inhibitors (e.g., anti-PD-1, anti-CTLA-4) enhance T cell activity by lifting inhibitory signals, which augments antitumor immunity but may also disrupt peripheral tolerance (190). This immune disinhibition may facilitate inappropriate responses to autoantigens or latent commensal antigens, including *C. acnes*. By disrupting Treg-mediated suppression, these agents may induce autoimmunity or DISRs in genetically or immunologically predisposed individuals.

IFN-α therapy, which promotes Th1 and Th17 responses, is associated with both secondary tuberculosis and DISRs, including *C. acnes*-associated sarcoidosis (63). This reactivation of latent microbes may occur when therapeutic immune modulation disturbs the delicate balance that maintains microbial latency (191).

Immune reconstitution inflammatory syndrome, which occurs in HIV patients initiating antiretroviral therapy, is characterized by exaggerated Th1 responses that may unmask latent infections such as MTB. Sarcoid-like granulomatous reactions, which clinically resemble sarcoidosis, have been documented in this setting (192).

Together, these observations suggest that immunomodulatory therapies may disrupt host control of latent pathogens, resulting in their reactivation and subsequent granulomatous inflammation in susceptible individuals. While reactivation of MTB is well established, the potential role of *C. acnes* in DISRs is increasingly recognized but warrants further investigation.

## 5 Conclusion

Sarcoidosis is a complex granulomatous disorder with an uncertain etiology. Emerging evidence implicates *C. acnes* as a potential endogenous antigen, particularly in genetically and immunologically susceptible individuals. This review highlights its contribution to immune dysregulation via persistent antigenic stimulation, Th1/Th17-skewed responses, and impaired Treg-mediated tolerance.

A defining feature of *C. acnes*-associated sarcoidosis is its capacity to evade immune clearance, thereby sustaining chronic inflammation. Although *C. acnes* is consistently detected within sarcoid granulomas and elicits robust Th1 responses, direct evidence of *C. acnes*-specific T cell responses remains insufficient. Further validation of pathogen-specific cellular immunity is critical to clarify its etiologic role across the disease spectrum, from self-limiting to progressive fibrotic forms.

Recent advances in immunohistochemistry and immune profiling have shown potential as tools for diagnosis and patient stratification. Development of *C. acnes*-targeted assays, including PAB antibody detection and Treg/Th17 immune signatures, may facilitate personalized therapeutic strategies.

Future interventions should focus on restoring immune tolerance and suppressing pathogenic T cell activation. Microbiota-directed therapies, such as intracellularly active antibiotics or probiotic modulation, also warrant further investigation. Stratifying patients based on *C. acnes*-associated immune phenotypes will be essential for implementing precision medicine. Standardization of biomarker assays and criteria for patient selection will also be pivotal for clinical translation.

Recognizing *C. acnes* as a central immunologic driver in sarcoidosis represents a paradigm shift in understanding this complex disorder. Bridging basic and clinical research will be essential for developing and implementing pathogen-targeted diagnostics and therapies that align with the principles of precision therapeutics. As our insights into host-microbe interactions deepen and our understanding of disease heterogeneity advances, integrating pathogen-specific strategies into individualized care holds promise for improving patient outcomes and shaping future research directions.
